# Zygomorphic flowers last longer: the evolution of floral symmetry and floral longevity

**DOI:** 10.1098/rsbl.2024.0082

**Published:** 2024-06-19

**Authors:** R. E. Stephens, R. V. Gallagher, M. Méndez, H. Sauquet

**Affiliations:** ^1^ School of Natural Sciences, Macquarie University, Ryde, New South Wales, Australia; ^2^ National Herbarium of NSW, Botanic Gardens of Sydney, Mount Annan, New South Wales, Australia; ^3^ Hawkesbury Institute for the Environment, Western Sydney University, Richmond, New South Wales, Australia; ^4^ Area of Biodiversity and Conservation, Universidad Rey Juan Carlos, Madrid, Spain; ^5^ Evolution & Ecology Research Centre, University of New South Wales, Sydney, New South Wales, Australia

**Keywords:** macroevolution, angiosperms, floral traits, floral evolution, pollination

## Abstract

Floral longevity, the length of time a flower remains open and functional, is a phylogenetically conserved trait that balances floral costs against the rate at which flowers are pollinated. Floral symmetry has long been considered a key trait in floral evolution. Although zygomorphic (bilaterally symmetric) flowers typically receive fewer floral visitors than actinomorphic (radially symmetric) flowers, it is yet to be determined whether this could be associated with longer floral longevity. Using newly collected field data combined with data from the literature on 1452 species in 168 families, we assess whether floral longevity covaries with floral symmetry in a phylogenetic framework. We find that zygomorphic flowers last on average 1.1 days longer than actinomorphic flowers, a 26.5% increase in longevity, with considerable variation across both groups. Our results provide a basis to discuss the ecological and evolutionary costs of zygomorphy for plants. Despite these costs, zygomorphy has evolved numerous times throughout angiosperm history, and we discuss which rewards may outweigh the costs of slower pollination in zygomorphic flowers.

## 1. Introduction

Floral longevity, the length of time a flower remains open and functional, is an important trait in pollination biology [1–3]. Optimal floral longevities balance flowering costs against the rate of pollen export and receipt [4,5]. Floral symmetry, whether flowers are bilaterally (zygomorphy) or radially (actinomorphy) symmetrical, has long been considered a key trait in floral evolution, potentially driving angiosperm speciation [6–9]. Zygomorphic flowers are considered more specialized with fewer floral visitors on average [10,11], but potentially more accurate cross-pollination [12,13].

A long-held but rarely tested hypothesis is that specialist zygomorphic flowers are more efficiently pollinated than generalist actinomorphic flowers [12,13]. Efficiency can mean that pollinators carry only conspecific, outcross pollen, and contact the stamens and stigma directly and precisely with it (the ‘complexity-constancy’ and ‘pollen position’ hypotheses [12]). Efficiency can also mean using minimal time to complete a task, however, and it remains an open question whether zygomorphic flowers achieve pollination more quickly than actinomorphic flowers. Experimental evidence has demonstrated direct connections between rates of pollen export and receipt and floral longevity [14]. Thus, the faster a species achieves successful pollen dispersal and/or receipt (hereafter ‘pollination’) on average, the shorter its floral longevity should be [4,14]. Floral longevity is thus a proxy measure for the speed of pollen dispersal and/or receipt, and the relationship between floral longevity and floral symmetry should provide insights into whether zygomorphic flowers achieve pollination more or less quickly than actinomorphic flowers.

Our study is the first to test the covariation between floral symmetry and floral longevity. We combine newly collected field data with data from the literature on floral longevity for 984 actinomorphic and 468 zygomorphic species across 168 angiosperm families. We consider two competing hypotheses. On the one hand, zygomorphic flowers typically receive fewer floral visitors than actinomorphic flowers, which could mean fewer pollinators, a slower rate of pollination and longer flowering overall to ensure pollination [4,11]. If this first scenario is the case, then we hypothesize that zygomorphic flowers would have longer mean floral longevity than actinomorphic flowers. On the other hand, if the fewer visitors to zygomorphic flowers are more effective pollinators, while the many visitors to actinomorphic flowers deposit heterospecific pollen or take rewards without dispersing pollen, zygomorphic species could exhibit shorter floral longevities overall [11,15,16]. If this second scenario is the case, then we predict that zygomorphic flowers would have shorter mean floral longevity than actinomorphic flowers. We assess these hypotheses in an evolutionary framework, given the strong phylogenetic signal of both floral symmetry and floral longevity, and provide one of the first descriptions of the evolutionary patterns of floral longevity across angiosperms as a whole. For completeness, we also assess whether the effect of symmetry on longevity is independent of the effect of latitude, given recent findings of longer floral longevity [3] and a higher frequency of actinomorphic species [17] at higher latitudes.

## 2. Methods

### (a) Floral longevity and symmetry data

Fieldwork to collect floral longevity data was conducted during peak flowering from July to November at three sites around Sydney, Australia (see electronic supplementary material, Notes S1 for details). Floral longevity data were collected following a protocol adapted from Wright *et al*.’s [18] methods for leaf lifespan, which allowed us to minimize survey effort and maximize the number of individual flowers and species surveyed. Briefly, at each site, we chose five individual plants ≥5 m apart of an equal number of actinomorphic and zygomorphic species in flower. On each plant, we tagged the base of an inflorescence and recorded the number of buds, anthetic flowers and senesced flowers in the inflorescence. Repeat surveys every 1–3 days (with visits every 2 days ideal, see electronic supplementary material, Notes S1) recorded the number of buds, anthetic flowers and senesced flowers in each inflorescence along with the exact time of survey until all flowers in the inflorescence had senesced. Any flowers damaged by florivory were excluded, and new inflorescences or plants were marked. Mean floral longevity was calculated for each inflorescence as the average time difference between the onset of flower anthesis and flower senescence for all flowers in that inflorescence. Our final field dataset included mean floral longevities for 17 actinomorphic and 17 zygomorphic species, with an average of 11.3 (s.e. = 0.7) flowers monitored per plant, and an average of 5.1 (s.e. = 0.1) plants per species (electronic supplementary material, NotesS1).

Species mean floral longevity from fieldwork data was combined with data compiled from more than 300 published floral longevity studies, as well as data from a recent global study of floral longevity [3]. Given many studies report only species mean floral longevity, we did not consider intraspecific variation in floral longevity. Floral longevity studies were only included where they recorded floral longevity on individual flowers under natural pollinator visitation, i.e. ‘realized’ floral longevity per Ashman & Schoen [4], as opposed to the maximum floral longevity possible when pollinators are excluded. Where floral longevity was reported as a range, we took the midpoint of the range, and we converted all longevity units into days. Species taxonomy was standardized to the World Checklist of Vascular Plants [19] and World Flora Online [20] using the Taxonomic Name Resolution Service [21]. We then calculated the mean floral longevity for each accepted species.

We assembled floral symmetry data from a combination of published studies [11,22], including Dressler *et al*. [23] via the TRY database [24] (*n* = 556), and manual scoring (*n* = 896). Symmetry was scored from a range of sources including species and higher taxa descriptions in floras, and images of herbarium or reliably identified fresh specimens. Our definition of floral symmetry followed the functional approach of Yoder *et al*. [11], primarily considering the symmetry of the perianth but also considering the symmetry of the androecium and gynoecium in marginal cases. Species with pseudanthia that combine actinomorphic and zygomorphic florets (e.g. Asteraceae, some Apiaceae) were scored according to the symmetry of the pseudanthium overall, given this is the unit of attraction that pollinators respond to. Symmetry was not scored for wind-pollinated taxa or other taxa with highly reduced perianths. Our final dataset included 984 actinomorphic and 468 zygomorphic species (total *n* = 1452) in 168 families and 802 genera.

For analyses of the effect of latitude, we obtained the latitude where each floral longevity study was completed either as it was reported in the paper or as estimated from the study location description’s geographic coordinates in Google Earth. In this analysis, we excluded studies where no clear location was described. We calculated species’ mean absolute latitude to match species mean longevity for each species, giving a final dataset for latitude analyses of 972 actinomorphic and 451 zygomorphic species (*n* = 1423).

### (b) Data analysis

c 4.3.0 [25] using the tidyverse collection [26] and functions from packages including phytools version 1.9-16 [27] and ape version 5.7-1 [28]. All data and analysis code are available at [29].

We used two seed plant phylogenies from Smith & Brown [30]: the GBOTB tree constructed from GenBank data for 79 881 taxa and the ALLOTB tree, which contains an additional 273 304 taxa from the Open Tree of Life. Species were matched to the ALLOTB and GBOTB phylogenies using a hierarchical approach: (i) direct matches of accepted names; (ii) direct matches of known synonyms; (iii) direct matches to closest species within the accepted genus; and (iv) matching to another species in a synonymous or sister genus. Any taxa that could not be reliably matched to a species in the phylogeny was excluded from analyses with that phylogeny, leaving 1433 species in ALLOTB analyses and 1187 species in GBOTB analyses.

Floral longevity was log-transformed for all phylogenetic analyses. We tested for phylogenetic signal using both the ALLOTB and GBOTB phylogenies, using Pagel’s *λ* [31] and Blomberg’s *K* [32] for floral longevity and using Caper [33] to calculate Fritz & Purvis’ *D* [34] for floral symmetry. To assess whether species mean floral longevity varies with floral symmetry, we ran phylogenetic generalized least squares (PGLS) regressions, considering all species in our data (ALLOTB analysis, *n* = 1433), species with reliable phylogenetic positioning (GBOTB analysis, *n* = 1187) and random subsampling of one species per genus to account for any potential sampling bias (50 random subsamples with ALLOTB phylogeny, *n* = 804). To assess whether the effect of symmetry on longevity is affected by latitude, we ran an additional PGLS model using the ALLOTB phylogeny with species mean absolute latitude and symmetry as fixed factors, both scaled to compare effect sizes.

## 3. Results

**Figure 1.**
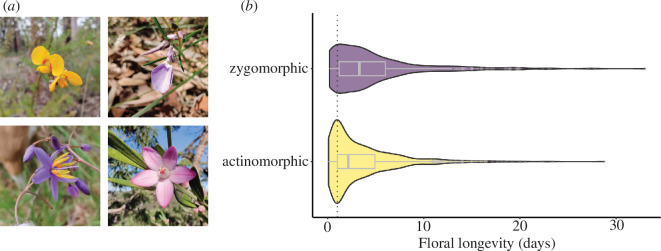
(*a*) Examples of zygomorphic (top) and actinomorphic (bottom) flowers monitored during floral longevity fieldwork. L–R *Dillwynia retorta* (Fabaceae, 3.6 days), *Hybanthus vernonii* (Violaceae, 14.1 days), *Dianella caerulea* (Asparagaceae, 1.1 days) and *Eriostemon australis* (Rutaceae, 8.4 days). Fieldwork data were combined with existing datasets to test for relationships between longevity and symmetry. (*b*) Floral longevity (as number of days) of zygomorphic (*n* = 468) versus actinomorphic (*n* = 984) flowering species. Box plots show median and interquartile range, violin plots show density distribution of data and dotted line indicates 1 day longevity.

Zygomorphic flowers had significantly longer mean floral longevities than actinomorphic flowers (table 1 and figure 1), supporting our first hypothesis. This trend was maintained across phylogenetic analyses, including sampling with the full ALLOTB phylogeny (*n* = 1433, *p* < 0.001), the GBOTB phylogeny (*n* = 1187, *p* < 0.001) and 48 out of 50 random subsamples of one species per genus (*n* = 804, *p* = 0.001–0.044; table 1). Zygomorphic flowers also had longer mean floral longevities than actinomorphic flowers when species mean absolute latitude was included in the analysis, although the effect of symmetry (scaled *β* = 0.14, *p* < 0.001) was slightly less than the effect of latitude (scaled *β* = −0.21, *p* < 0.001). Mean floral longevities for actinomorphic flowers ranged from 3.4 to 3.6 days across all sampling and zygomorphic flowers from 4.5 to 5 days (table 1).

**Table 1.** Key results from phylogenetic generalized least squares (PGLS) regressions, including analysis of 50 random subsamples of one species per genus.

phylogeny	sampling	sample size			mean floral longevity		PGLS *p*‐value
		total	zyg.	actin.	zyg.	actin.	
ALLOTB	all	1433	458	975	4.7	3.6	<0.001
GBOTB	all	1187	366	821	4.9	3.6	<0.001
ALLOTB	one species per genus	804	234–243	561–570	4.5–5.0	3.4–3.5	0.001–0.058

Both actinomorphic and zygomorphic flowers showed a considerable range in floral longevity around the means (figure 1, electronic supplementary material, figure S1). The longest-lived zygomorphic flower in our data, *Telipogon peruvianus* (Orchidaceae), maintained individual flowers for 33 days, followed by the longest-lived actinomorphic flower *Siparuna muricata* (Siparunaceae) at an average of 28.75 days. Most flowers were relatively short lived, however, with many described as flowering for 1 day or less, particularly for actinomorphic flowers (figure 1).

**Figure 2.**
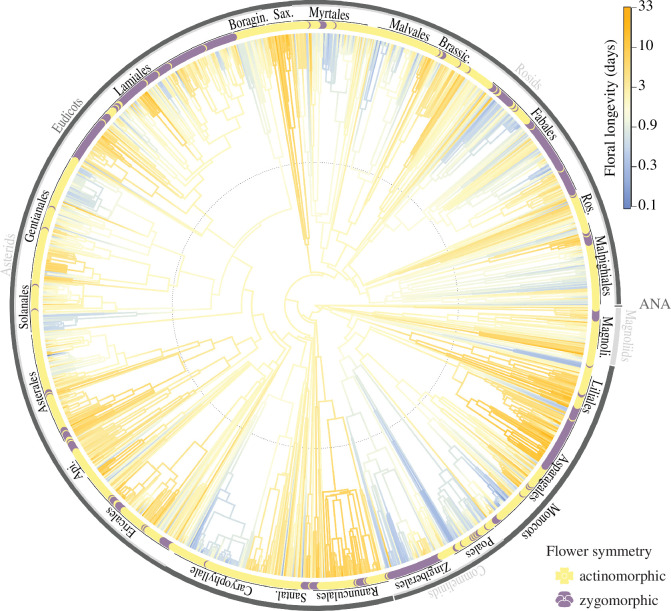
Evolutionary tree of all species matched the Smith & Brown [30] ALLOTB angiosperm phylogeny (*n* = 1433). Branch colour gradient shows species mean floral longevity modelled on a log scale to highlight values below and above 1 day, points at tips indicate species floral symmetry, and major angiosperm clades and orders are labelled. ANA = Amborellales, Nymphaeales and Austrobaileyales.

Floral symmetry and floral longevity both showed moderate to strong phylogenetic signal (symmetry *D* = −0.3, longevity *λ* = 0.8, *K* = 0.1, all *p* ≤ 0.001). The phylogenetic distribution of floral longevity and floral symmetry can be seen in figure 2 (electronic supplementary material, figure S1 , electronic supplementary material, tables S4–S6 for more details). Orders with the longest-lived flowers on average include Liliales (mean = 9.3 days) and Saxifragales (9.3 days), and the shortest include Poales (1.1 days) and Boraginales (1.9 days). Mean floral longevity for the angiosperms as a whole was 3.9 days (s.e. = 0.1, *n* = 1452).

## 4. Discussion

Zygomorphic flowers have long been thought to receive more efficient pollination. Here, we find that zygomorphic flowers remain open and functional for longer than actinomorphic flowers, suggesting that effective pollen dispersal and receipt takes longer to occur on average in zygomorphic flowers (figure 1*b*). This result was robust to different levels of phylogenetic sampling and resolution, suggesting an evolutionary correlation between the symmetry and longevity of flowers. Indeed, many primarily actinomorphic families have mean floral longevities less than 1 day (e.g. Convulvulaceae, Cactaceae and Cistaceae) while some zygomorphic families have floral longevity greater than 10 days (e.g. Lentibulariaceae, Orchidaceae, figure 2, electronic supplementary material, figure S1, electronic supplementary material, tables S5 and S6). Our results suggest a key cost of zygomorphy, as zygomorphic flowers have to be maintained for longer than actinomorphic flowers.

The increased floral longevity of zygomorphic flowers may be related to their lower rate of floral visitation overall [11]. Zygomorphic flowers appear to play a ‘waiting game’, maintaining anthesis for longer to increase their chances of attracting a visit from the smaller pool of potential pollinators able to access their flowers. This maintenance would have costs, not least the water needed to maintain petal turgor and carbon for replenishing nectar supplies [4,5,35]. The trade-off may be that zygomorphy, along with related traits such as horizontal floral orientation or floral tubes, encourages more precise pollination with less interspecific pollen transfer [36,37]. Such pollination precision may be particularly advantageous for flowers that are vulnerable to pollen interference from heterospecific pollen [15].

Zygomorphy has evolved at least 154 times from actinomorphic ancestors and is currently found in at least 32 orders and 110 families of angiosperms [13,17,22]. The macroevolution of floral longevity has been less well described to date, but our phylogenetic exploration confirms previously noted patterns (e.g. shorter floral longevity in Commelinaceae and longer in Orchidaceae, electronic supplementary material, figure S1 and [2]). While greater sampling is needed to properly reconstruct the evolution of floral longevity, our initial findings suggest that floral longevity is generally only a few days across the angiosperms (electronic supplementary material, figure S1, mean = 3.9 days), with a distinct phylogenetic structure of clades with shorter (e.g. Convolvulaceae) and longer (e.g. Orchidaceae) floral longevity (figure 2, electronic supplementary material, tables S4–S6). This is reflected in the strong phylogenetic signal found for floral longevity (*λ* = 0.8).

Aside from the influence of symmetry found here, there is a great deal of variability in floral longevity that remains unexplained (figure 1*b*). Some of this variability may reflect variability in rates of floral visitation to actinomorphic versus zygomorphic flowers, given some actinomorphic flowers can be quite specialized in their pollination (e.g. within Apocynaceae [38]) while some zygomorphic flowers are visited by many pollinators [11,39]. Floral symmetry is by no means the only floral trait involved in pollination specialization, and other floral traits such as floral tubes or floral orientation could mediate the effect of symmetry on longevity by affecting rates of pollinator visitation [40]. Reproductive traits such as dichogamy, self-incompatibility and cleistogamy may also play a role in the relationship between floral longevity and floral symmetry, given these traits can affect the speed at which a flower achieves its function and may be unevenly distributed between zygomorphic and actinomorphic species [22,41,42]. Self-compatibility, for example, is more common in zygomorphic flowers yet does not necessarily lead to increased self-pollination where zygomorphy impedes self-pollen deposition [41]. Floral symmetry could thus mediate the relationship between self-incompatibility and floral longevity, which has been hypothesized but not yet demonstrated [3]. Analyses that consider the combined effects of multiple floral and reproductive traits may lead to a more nuanced view of the relationship between floral longevity and floral symmetry in future research.

Despite its strong phylogenetic signal, floral longevity can be responsive to environmental conditions, and other correlates of floral longevity include latitudinal distribution and temperature [3]. Although the effect of symmetry on longevity was robust to the effect of latitude on longevity, many other factors may mediate this relationship on a global scale. Although Song *et al*. [3] found no evidence for a relationship between flower size and floral longevity, these key features of the floral display may be mediated by the trade-off between flower size and flower number or environmental context and bear further investigation [2,5,43,44]. For example, plants may produce large, long-lived floral displays of many small flowers, each of which opens for only a short time but is quickly replaced by another flower. In this way, selection for floral longevity could operate at the whole plant level, rather than that of the individual flower, contributing to the variability of floral longevities across the angiosperms as a whole.

Here, the correlation with longer floral longevity reveals a potential cost of zygomorphy for flowers and plants. Future research into flower economics may uncover further evolutionary correlates of floral longevity. Further study could determine, for example, whether zygomorphic flowers spend less than comparable actinomorphic flowers on resources such as pollen, nectar or water in compensation for their extended lifespan. Flower size and number trade-offs, and their interaction with the trade-off between floral longevity and floral symmetry, also bear investigation. Flower economics can thus expand our understanding of this critical aspect of plant reproduction, and the evolutionary compromises shaping the diversity of flowers around us.

## Data Availability

Full data, data processing and analysis code are available in a GitHub repository archived via Zenodo at [29]. Electronic supplementary material is available online [45].
